# Population-Based Screening for Endometrial Cancer: Human vs. Machine Intelligence

**DOI:** 10.3389/frai.2020.539879

**Published:** 2020-11-24

**Authors:** Gregory R. Hart, Vanessa Yan, Gloria S. Huang, Ying Liang, Bradley J. Nartowt, Wazir Muhammad, Jun Deng

**Affiliations:** ^1^ Department of Therapeutic Radiology, Yale University, New Haven, CT, U.S.A; ^2^ Department of Statistics and Data Science, Yale University, New Haven, CT, U.S.A; ^3^ Department of Obstetrics, Gynecology and Reproductive Sciences, Yale University, New Haven, CT, U.S.A

**Keywords:** endometrial cancer, cancer screening, early detection, machine learning, statistical biopsy

## Abstract

Incidence and mortality rates of endometrial cancer are increasing, leading to increased interest in endometrial cancer risk prediction and stratification to help in screening and prevention. Previous risk models have had moderate success with the area under the curve (AUC) ranging from 0.68 to 0.77. Here we demonstrate a population-based machine learning model for endometrial cancer screening that achieves a testing AUC of 0.96.

We train seven machine learning algorithms based solely on personal health data, without any genomic, imaging, biomarkers, or invasive procedures. The data come from the Prostate, Lung, Colorectal, and Ovarian Cancer Screening Trial (PLCO). We further compare our machine learning model with 15 gynecologic oncologists and primary care physicians in the stratification of endometrial cancer risk for 100 women.

We find a random forest model that achieves a testing AUC of 0.96 and a neural network model that achieves a testing AUC of 0.91. We test both models in risk stratification against 15 practicing physicians. Our random forest model is 2.5 times better at identifying above-average risk women with a 2-fold reduction in the false positive rate. Our neural network model is 2 times better at identifying above-average risk women with a 3-fold reduction in the false positive rate.

Our machine learning models provide a non-invasive and cost-effective way to identify high-risk sub-populations who may benefit from early screening of endometrial cancer, prior to disease onset. Through statistical biopsy of personal health data, we have identified a new and effective approach for early cancer detection and prevention for individual patients.

## Introduction

Endometrial cancer is the fourth most common cancer among women (Howlader et al., 2017). Symptoms such as bleeding or spotting often manifest early in the disease, resulting in the early detection of most cancers and a relatively high 5-years survival rate of 82% (American Cancer Society, 2017). The standard method for detecting endometrial cancer is endometrial biopsy, although transvaginal ultrasounds are sometimes used for detection as well (Smith et al., 2001; Smith et al., 2018). Screening recommendations from the American Cancer Society (ACS) have remained constant since 2001 (Smith et al., 2018). Women with average or elevated risk are not recommended to get screened; instead, they should discuss with their doctor about the risks and symptoms of endometrial cancer at the onset of menopause. For very high-risk women such as those with Lynch syndrome, a high likelihood of being a mutation carrier, or families with suspected autosomal-dominant predisposition to colon cancer, ACS recommends annual screening (Smith et al., 2001).

While the 5-years survival rate for endometrial cancer is high, incidence and death rates of endometrial cancer have increased from 2010 (Howlader et al., 2017) and are expected to continue to increase (Arnold et al., 2015). It is expected that endometrial cancer will soon surpass ovarian cancer as the leading cause of gynecological cancer death. This has led to academic interest in improving endometrial cancer detection and prevention.

Two previous studies have been carried out to predict endometrial cancer risk (Pfeiffer et al., 2013; Hüsing et al., 2016). Both studies use traditional epidemiological models and non-invasive data for decision-making on targeted screening and preventive procedures. Hüsing *et al* trained a model on a dataset of 201,811 women (mostly aged 30–65 years), with 855 positive cases of endometrial cancer (0.4%). This model achieved an AUC of 0.77 (Hüsing et al., 2016). Pfeiffer et al’s model (Pfeiffer et al., 2013), which produced an AUC of 0.68, was trained on the same PLCO dataset that we used, in addition to the NIH-AARP dataset. Their full dataset had a total of 304,950 women with 1,559 positive cases of endometrial cancer (0.51%). Noting the moderate performance of endometrial risk stratification models that were previously created (Hüsing et al., 2016), and the promising results of our previous work in using machine learning for cancer risk stratification (Hart et al., 2018; Roffman et al., 2018a; Roffman et al., 2018b; Muhammad et al., 2019), we decided to develop machine learning models to achieve greater performance in predicting endometrial cancer risk. We were able to surpass the performance of both these models with an AUC of 0.96.

Finally, a recent review suggests that a risk prediction model that divides the population up into low-, medium-, and high-risk groups would be useful for developing tailored cancer prevention strategies for each patient (Kitson et al., 2017). Such a model can help clinicians target high-risk populations, for whom clinicians could suggest interventions to modulate endometrial cancer risk, such as dietary and exercise changes, progestin or anti-estrogen therapy, insulin-lowering therapy, and scheduled endometrial biopsies. This is why we further applied our machine learning model to stratify patients into low-, medium, and high-risk groups. We compared our model’s performance on the 3-tier risk stratification with physicians’ judgment and achieved promising results.

## Methods

### The PLCO Dataset

In this study we developed our machine learning models based on the Prostate, Lung, Colorectal, and Ovarian Cancer Screening Trial (PLCO) dataset (Kramer et al., 1993). PLCO was a randomized, controlled trial investigating the effectiveness of various screenings for prostate, lung, colorectal, and ovarian cancers. It was a prospective study that enrolled participants from November 1993 through July 2001. Participants were between 55 and 75 years old. Shortly after enrollment, participants completed a baseline survey detailing their health history and current health condition. They were then followed until they were diagnosed with cancer or died, or when 13 years had passed. From the PLCO dataset, we sub-selected the 78,215 female participants for whom we have data on whether they developed endometrial cancer within 5 years of enrolling in the PLCO trial. 961 of these females developed endometrial cancer within five years of enrolling. This gave 77,254 non-cancer cases (98.8%) and 961 cancer cases (1.2%) on which we train our model. For full details about this data and its collection see Kramer et al., 1993.

With authorization from the National Cancer Institute (NCI) to access PLCO trial data (PLCO-365), we used the following inputs for our model: age (Howlader et al., 2017), BMI(Renehan et al., 2008; Crosbie et al., 2010), weight (20 years, 50 years, present) (Hosono et al., 2011; Aune et al., 2015), race (Howlader et al., 2017), smoking habits (Zhou et al., 2008), diabetes (Anderson et al., 2001), emphysema, stroke, hypertension (Aune et al., 2017), heart disease, arthritis (Parikh-Patel et al., 2009), another cancer, family history of breast, ovary, and endometrial cancer, ovarian surgery (Dossus et al., 2010), menarche age (Dossus et al., 2010), parity (Dossus et al., 2010), use of birth control (Dossus et al., 2010), and age at menopause (Dossus et al., 2010). Many of these inputs, such as BMI, diabetes and family history, were selected because they correlate strongly with endometrial cancer incidence (Anderson et al., 2001; Dossus et al., 2010; Aune et al., 2015) and were also used as inputs in other works on endometrial cancer risk prediction (Pfeiffer et al., 2013; Hüsing et al., 2016; Kitson et al., 2017). Other factors, such as smoking habits, emphysema and heart disease, were included because they contributed to good performance in our past works. (Hart et al., 2018; Roffman et al., 2018a; Roffman et al., 2018b; Muhammad et al., 2019). There are other known risk factors such as Hereditary Non-polyposis Colorectal Cancer (HNPCC) or Lynch Syndrome which would be good to include in a model but are not in the PLCO dataset. All inputs were scaled to within the range of [0, 1].

To evaluate the different algorithms, we randomly split the dataset 70%/30% into training and testing sets, keeping the proportion of those with and without cancer constant between the two datasets. The final model was trained on the full training set and evaluated on the testing set. This gives our model a Transparent Reporting of a multivariable prediction model for Individual Prognosis Or Diagnosis (TRIPOD) level 2a of robustness (Collins et al., 2015).

### Machine Learning Algorithms

In creating our risk prediction models, we trained algorithms that produce continuous output from 0 to 1, which indicated the probability that a woman would develop endometrial cancer within five years since the input data was gathered. The algorithms we used were: logistic regression (LR), neural network (NN), support vector machine (SVM), decision tree (DT), random forest (RF), linear discriminant analysis (LDA), and naive Bayes (NB) (Bishop, 2006). The logistic regression was fit using the NN code with 0 hidden layers. The NN was fit using the in-house MATLAB code we developed for previous works (Hart et al., 2018; Roffman et al., 2018a; Roffman et al., 2018b; Muhammad et al., 2019). It was a multilayer perceptron consisting of two hidden layers with 12 neurons each and a logistic activation function. We then used the built-in MATLAB function “fitrsvm” with a Gaussian kernel to fit the SVM, and we used the function “fitctree” to create the decision tree. The random forest was fit with the built-in MATLAB function “TreeBagger” with 50 trees. The LDA was fit using the built-in MATLAB function “fitcdiscr,” with “discrimType” set to “diaglinear”. Lastly, the NB was fit using the built-in MATLAB function “fitcnb,” with “OptimizeHyperparameters” set to “auto”. For “fitctree”, “fitcdiscr”, and “fitcnb,” the “score” from the “predict” function was used to get continuous output, akin to that returned by the LR, NN, and SVM. We used these six algorithms because they are well-established machine learning techniques.

In selecting the algorithm for the final model(s), we used 10-fold cross-validation within the training and testing sets to determine the mean AUC of each algorithm. We identified the two models that achieved the highest testing mean AUCs between training and testing performance. These two models were then trained on the full training set and evaluated on the testing set. Afterward, for each of the two best models, we selected a threshold for determining the sensitivity, specificity, positive predictive value (PPV) and negative predictive value (NPV), by maximizing the sum of the training sensitivity and specificity, i.e., maximizing the balanced accuracy.

### Risk Stratification

Once we selected the two best models, we used them to stratify the population into below, at, or above-average risk, to facilitate a comparison of our models’ prediction to physicians’ judgment in the clinic. Specifically, in selecting the boundaries based on the training data, we considered the bottom 15.9% of risks as below average, the top 15.9% of risks as above average, and the middle 68.2% as average risks.

### Human Intelligence (HI) vs. Artificial Intelligence (AI)

For comparison of the models’ prediction against physicians’ judgment, we created an online survey (https://yalesurvey.ca1.qualtrics.com/jfe/form/SV_3TVh1XP27eaktud) with a sub-data set of 100 women from our original dataset. The survey presented the information used by our model to physicians and asked them to rate each woman as below, at, or above-average for endometrial cancer risk. Clinicians were given no instructions on how to classify individuals, so that we would get results representative of what would happen in practice. In an effort to get high-quality data, we limited the length of the survey by only showing each physician a random subset of 20 of the 100 women. The answers from the various physicians were aggregated and averaged for each woman. We then used our model to stratify the same group of women. We invited physicians from Yale, Harvard, and University of Michigan Departments of Obstetrics, Gynecology, and Reproductive Science/Biology, as well as primary care physicians from INOR Cancer Hospital (Abbottabad, Pakistan) and Yale Health Center to participate. We received usable responses from 15 physicians.

## Results

We evaluated seven different algorithms: logistic regression (LR), neural network (NN), support vector machine (SVM), decision tree (DT), random forest (RF), linear discriminant analysis (LDA), and naïve Bayes (NB). Table 1 presents the mean average area under the receiver operating characteristic (ROC) curve (AUC) with one standard deviation, from the 10-fold cross-validation on both the training and testing datasets. The training AUCs range from 0.68 to 0.99 and the testing AUCs range from 0.68 to 0.95. There is no significant difference in the training and testing performance for four of the algorithms (LR, NN, LDA and NB), but SVM, DT, and RF have a significant drop in performance going from training to testing. The highest testing performance was for the RF, although NN and RF testing performance are within one standard deviation of each other. For the remainder of this paper we will be focusing on the random forest and neural network models because these two models achieved the highest mean testing AUCs during cross-validation.

**TABLE 1:** Mean AUC (standard deviation) over 10 cross-validation folds for the seven algorithms tested.

	LR	NN	SVM	DT	RF	LDA	NB
Training	0.68 (0.11)	**0.89 (0.05**)	0.99 (0.00)	0.98 (0.00)	**0.99 (0.01)**	0.81 (0.00)	0.72 (0.12)
Testing	0.68 (0.10)	**0.88 (0.07)**	0.80 (0.03)	0.85 (0.04)	**0.95 (0.01)**	0.81 (0.03)	0.72 (0.12)

Selecting the random forest and neural network as the top models, we then trained them on the full training dataset and evaluated them on the testing dataset. When calculating the models’ performance on both the training and testing datasets, we calculated 95% confidence intervals of the AUC, sensitivity, specificity, PPV, and NPV (Hanley and McNeil, 1982).

**FIGURE 1:**
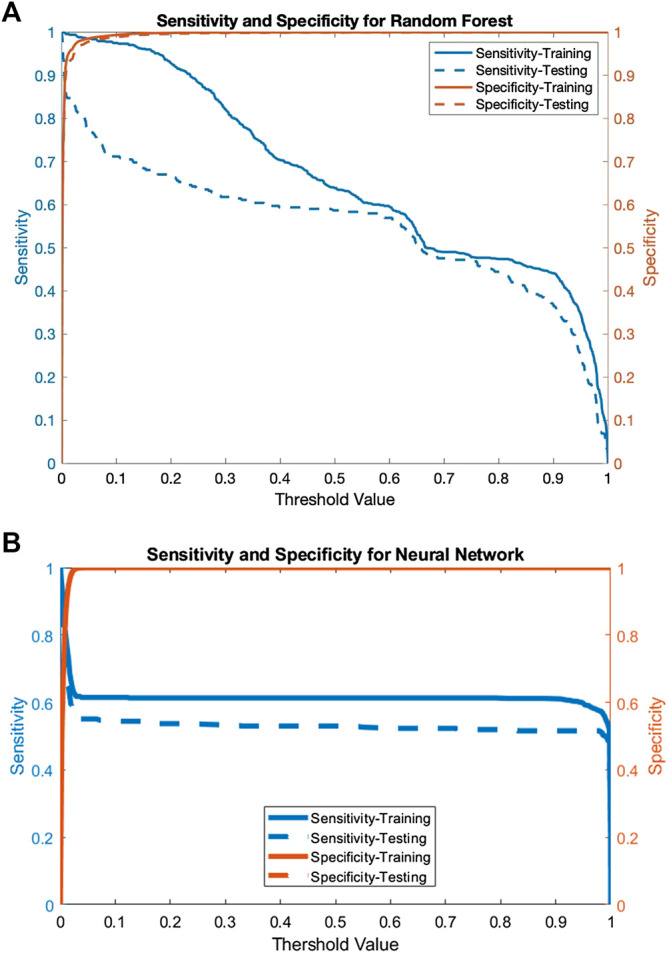
**A)** The sensitivity and specificity of the random forest for both the training and testing data as a function of the threshold value and **(B)** The sensitivity and specificity of the neural network for both the training and testing data as a function of the threshold value.

Figure 1A shows the sensitivity and specificity as a function of the decision threshold for the random forest on both the training and testing datasets. The same is done for the neural network in Figure 1B. For the random forest, the sensitivity decreases as the threshold value increases, while the specificity is above 99.9% on both the training and testing datasets. For the neural network model, the sensitivity hovers around 60% and the specificity remains above 99.9% for most threshold values on both the training and testing datasets. Given a threshold value that maximizes the sum of the training sensitivity and specificity, the random forest model’s sensitivity is 98.4% for the training set and 75.7% for testing. The specificity is 98.9 and 98.3% for training and testing respectively. The neural network model’s sensitivity is 77.2% for the training set and 67.7% for testing. The specificity is 91.2 and 91.1% for training and testing, respectively.

**FIGURE 2:**
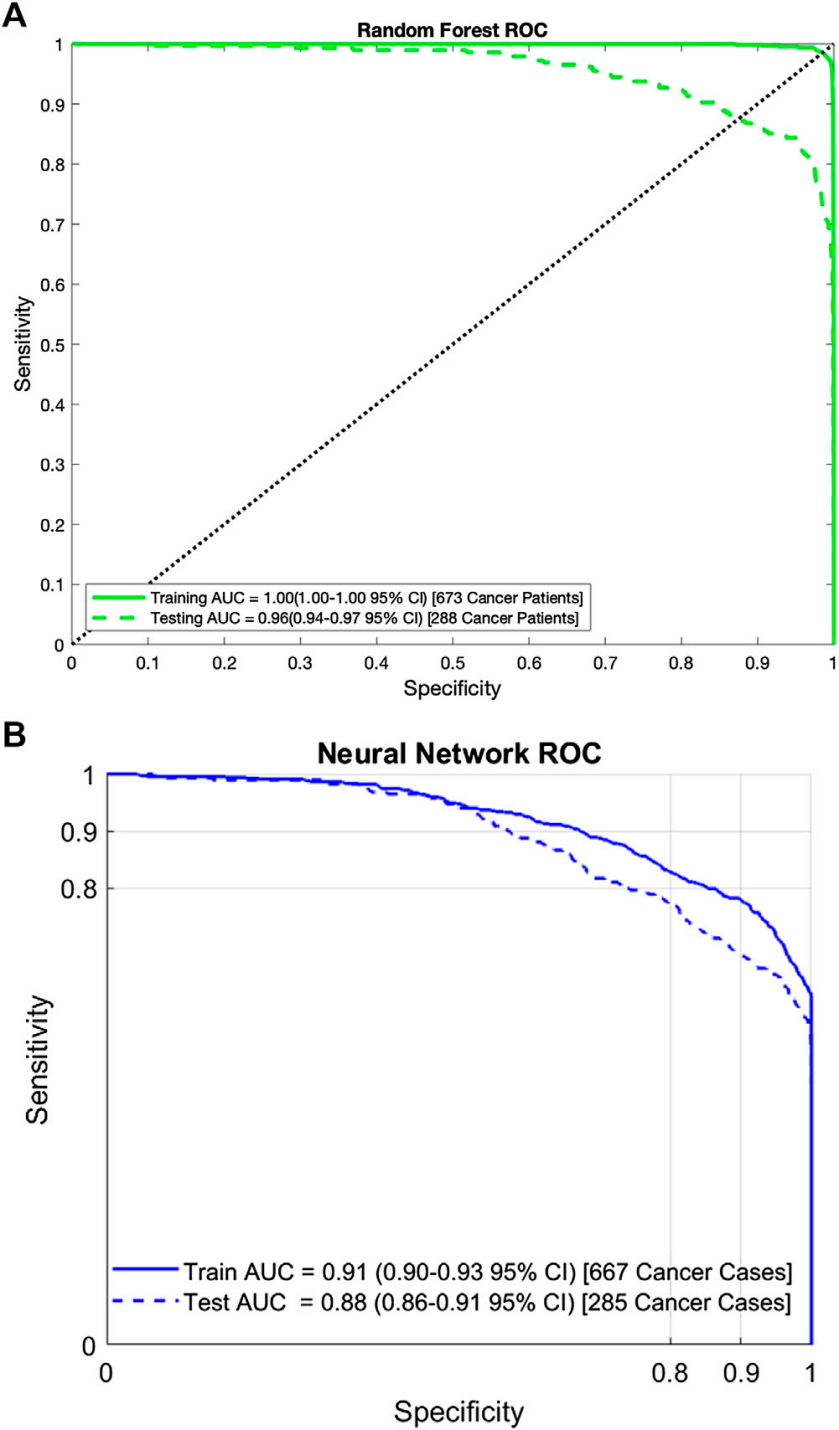
**A)** Area under the ROC curve for the random forest on both the training and testing data. Similar performance on both datasets indicates that the random forest has no overfit and **(B)** Area under the ROC curve for the neural network on both the training and testing data. Similar performance on both datasets indicates that the neural network has no overfit.

Using the sensitivity, specificity, and prevalence of endometrial cancer, we calculate the PPV and NPVs as well. For random forest, the PPV is 28.2 and 16.3% for the training and testing datasets respectively. The NPV is 99.9 and 99.9% for training and testing respectively. For the neural network, the PPV is 3.8 and 3.3%, and the NPV is 99.9 and 99.8%, for the training and testing datasets respectively. The ROC curves for the random forest and neural network are shown in Figures 2A,B. For the random forest, the AUC for training and testing are, respectively, 0.99 (95% CI: 0.99–1.00) and 0.96 (95% CI: 0.94–0.97). For the neural network, the AUC for the training data is 0.91 (95% CI: 0.90–0.93) and for testing it is 0.88 (95% CI: 0.86–0.91).

**FIGURE 3:**
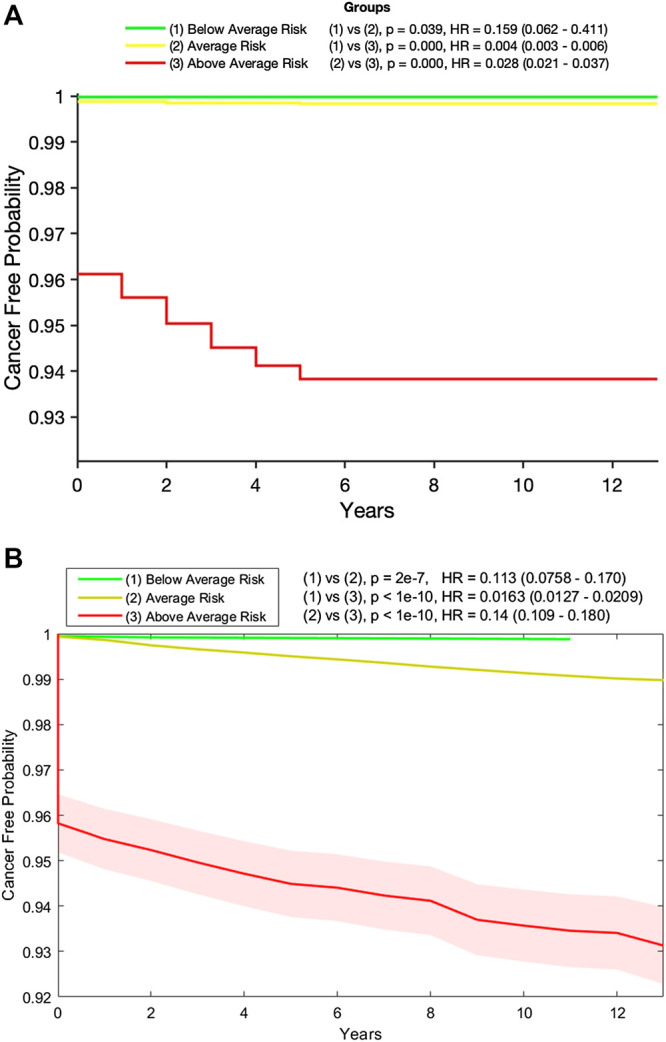
**(A)** Kaplan-Meier plot of the below- (green), at- (yellow), and above- (red) average risk groups created from the testing data by our random forest model. Also shown are the *p*-value and hazard ratio (HR) between each group. Those in the above-average risk group clearly have the highest chance of developing cancer and **(B)** Kaplan-Meier plot of the below- (green), at- (yellow), and above- (red) average risk groups created from the testing data by our neural network model with 95% confidence intervals (shaded). Also shown are the *p*-value and hazard ratio (HR) between each group. Those in the above-average risk group clearly have the highest chance of developing cancer.

Following the recommendation of Ref 8, we used the random forest and neural network models to create a 3-tiered risk stratification scheme. Based on the risk boundaries selected using the training data, we stratified the testing data into three groups: below, at, and above-average risk. Figures 3A,B show Kaplan-Meier plots for these three groups over the full 13 years they were followed. The figures clearly show that women classified as above-average risk have the highest chance of developing endometrial cancer. This is supported further by the hazard ratio (HR) between the above-average group and the two other groups.

In fact, as shown in Table 2, 90.3% of those in the testing set who developed endometrial cancer during the next 5 years were labeled by the random forest model as above-average risk and 15.7% of those who did not develop cancer were labeled as below-average risk. The incidence rates in the below-average, average, and above-average risk groups are 0.03, 0.17, and 6.17%, respectively. Similar performances were observed for the neural network as shown in Table 3

**TABLE 2:** Stratifying the testing data into three risk groups by the random forest.

	Below average risk		Averagerisk		Above average risk	
	Number	%	Number	%	Number	%
Cancer	1	0.3	27	9.4	260	90.3
No cancer	3,628	15.7	15,592	67.3	3,956	17.1

The percentages in each row sum up to 1.

**TABLE 3:** Stratifying the testing data into three risk groups by the neural network.

	Below average risk		Averagerisk		Above average risk	
	Number	%	Number	%	Number	%
Cancer	3	1.0	76	26.7	206	72.3
No cancer	3,705	16.0	15,920	68.7	3,553	15.3

The percentages in each row sum up to 1.

Tables 4, 5 show the comparison of our models with practicing clinicians in assessing risk for 100 women. In the below-average risk population, the physicians identified 2.8 times as many women who did not develop cancer as being below-average risk, compared to our random forest model (39.5 vs. 14.0%), and 1.65 times as many as our neural network model (39.5 vs. 24.0%). However, the physicians misidentified twice as many women who did not develop cancer as being high risk, compared to the random forest model (27.9 vs. 14.0%), and 3.5 times as many compared to the neural network (27.9 vs. 8.0%). Furthermore, our model was much better than physicians at aptly stratifying patients who would develop endometrial cancer. Physicians misidentified 22% of those who did develop cancer as having below average risk, whereas our random forest and neural network models predicted none. Additionally, compared to physicians’ predictions, our random forest model identified 2.5 times as many women who did develop cancer (94.0 vs. 38.0%) as above-average risk, and our neural network model identified almost twice as many as the physicians did (70.0 vs. 38.0%). Finally, there is a large inter-observer variability on the physicians’ assessments, while our models return the same predictions every time.

**TABLE 4:** Random forest model vs. physician stratification of 50 women with cancer (ground truth positives) and 50 women without cancer (ground truth negatives) into below-, at-, or above-average risk groups.

	Below average risk		Average risk		Above average risk	
	Model	Physicians	Model	Physicians	Model	Physicians
Ground truth positives	0.0	22.0% (17%)	6.0	40.0% (16%)	94.0	38.0% (24%)
Ground truth negatives	14.0	39.5% (22%)	72.0	32.6% (16%)	14.0	27.9% (20%)

Inter-observer variability for the physicians is captured by a standard deviation in parenthesis.

**TABLE 5:** Neural network model vs. physician stratification of 50 women with cancer (ground truth positives) and 50 women without cancer (ground truth negatives) into below-, at-, or above-average risk groups.

	Below average risk		Average risk		Above average risk	
	Model	Physicians	Model	Physicians	Model (%)	Physicians
Ground truth positives	0.0	22.0% (17%)	30.0	40.0% (16%)	70.0	38.0% (24%)
Ground truth negatives	24.0	39.5% (22%)	68.0	32.6% (16%)	8.0	27.9% (20%)

Inter-observer variability for the physicians is captured by a standard deviation in parenthesis.

## Discussion

We created seven different models to predict the probability of an individual woman developing endometrial cancer in five years based on readily available personal health data. Of these seven models we found that the random forest model performed best in terms of testing AUC, and the neural network performed second best. We then used both models to stratify the population into three risk categories. The above-average risk category captured the majority of those who developed cancer in five years. This above-average risk population could benefit from regular screening procedures such as endometrial biopsy and/or transvaginal ultrasounds.

Of our seven models, logistic regression and naive Bayes performed the worst and had the most variation between cross-validation folds. We think that the relatively poor performance of logistic regression and naive Bayes is due to the lack of interaction terms in these models. Without interactions between the input factors, these models have no advantage over traditional epidemiological models. A neural network with at least one hidden layer allows for mixing of the input parameters, which may explain its outperforming the other algorithms we tested.

Four of the models (LR, NN, LDA and NB) generalized well with similar training and testing AUCs, while SVM and DT overfit on the training data. Even though SVM, DT and RF achieved near-perfect AUC on the training data, they still performed better on the testing data than previous works; a phenomenon we also saw with lung cancer (Hart et al., 2018). The neural network achieved an AUC of 0.88 on both the 10-fold cross-validation and the testing set. The random forest achieved a testing AUC of 0.96. Both our random forest and neural network models significantly outperformed two previous risk prediction models, including the model introduced by Pfeiffer et al which achieved an AUC of 0.68 (Pfeiffer et al., 2013). This improvement is particularly interesting because Pfeiffer et al trained their model on not only the PLCO data, but also data from the National Institutes of Health-AARP Diet and Health Study. Although our model outperforms their model, theirs is more robust since it has been validated on an external data set, making it TRIPOD level 3 compared to our level 2a. Another previous work, by Hüsing *et al*, achieved an AUC of 0.77 (Hüsing et al., 2016). Their improvement was made by explicitly adding interaction terms to the epidemiological model. They used cross-validation making it TRIPOD level 1b. We are seeking access to the datasets used in these other works as external testing on our model.

With our random forest and neural network models outperforming previous works, we turn our attention to comparing our models with clinical judgment. The ultimate goal of this and our previous work is to create a risk prediction tool that can support physicians in their clinical decision-making about cancer prevention and screening for individuals prior to disease onset. In stratifying 100 women into below-, at-, and above-average risk groups, the physicians’ true negative rate in the below-average group was 1.6 times better than that of our neural network model (39.5 vs. 24.0%). However, physicians’ judgment resulted in a worse false negative rate in the below-average group (22 vs. 0%) and lower true positive rate in the above-average group, compared to both our random forest and neural network models. Thus, we have shown that our machine learning models are better than practicing physicians at identifying high-risk above average risk women.

While current guidelines only recommend screening for very high-risk women, our models may be capable of identifying a larger population who would benefit from screening. In fact, when stratifying the population based on stricter criteria (Hart et al., 2018; Roffman et al., 2018a; Roffman et al., 2018b; Muhammad et al., 2019) than what was used in this paper, our neural network model identifies a high-risk group in which 47% of women developed endometrial cancer within 5 years, among whom most developed the cancer under a year (data not shown). In addition to informing women and their physicians in their discussion of the potential pros and cons of screening, our models can help prompt life-style changes and other preventive measures or intervention (see Arnold et al., 2015). Admittedly, the downside to our models for this application is that understanding the contribution of individual input factors to the overall risk is not intuitive. So, while the current model can stratify the population and suggest the above-average risk group to participate in preventive strategies, it does not offer much help in deciding which strategies (e.g., diet and exercise, progestin or anti-estrogen therapy, and insulin-lowering therapy etc.) would be most effective. We will carry out this study in our future works. Nevertheless, our machine learning approach shows great promise in aiding early detection of endometrial cancer, as the approach yields high-accuracy predictions based solely on personal health information prior to disease onset, without need for any invasive or costly procedures like endometrial biopsy or transvaginal ultrasounds. Furthermore, it could be integrated into existing electronic medical record systems, giving risk predictions directly to primary care physicians when they see patients.

Compared with clinical judgment, the strong performance of our models, combined with other strongly discriminatory models for non-melanoma skin cancer (Roffman et al., 2018a), prostate cancer (Roffman et al., 2018b), lung cancer (Hart et al., 2018), and pancreatic cancer (Muhammad et al., 2019), presents a real opportunity to perform a “statistical biopsy” on individuals prior to disease onset. Analogous to traditional biopsy, which analyzes cells from a specimen, and the recently developed liquid biopsy, which evaluates circulating DNA from a blood sample to diagnose cancer, our machine learning approach to cancer prediction is essentially a statistical biopsy that mines personal health data of an individual for early cancer detection and prevention. Different from traditional biopsy and liquid biopsy, statistical biopsy seeks to decipher the invisible correlations and inter-connectivity between multiple medical conditions and health parameters via statistical modeling. By mining personal health data via statistical biopsy, it is possible to generate a holistic profile of an individual’s risk for a variety of cancer types, with little cost in time or money and no side effects. Furthermore, if integrated into a modern electronic medical record (EMR) system, statistical biopsy may help inform preventive interventions and/or screening decisions in real time. As we test our models on external datasets and expand the types of cancer covered, we hope to build a comprehensive model available to primary care physicians worldwide, allowing for statistical biopsies during routine clinical care for the general public.

## Conclusion

In this work we construct machine learning models to predict the five-year risk of developing endometrial cancer for individual women based solely on personal health data, without any genomic or imaging biomarkers, or invasive procedures. We test seven different algorithms and find that the random forest performs optimally and outperforms previous models. We further demonstrate that the random forest is superior to the 15 physicians in stratifying the population into three risk groups, with a 2.5-fold increase in true positive rate, 2-fold reduction in false positive rate, and reduction to zero in false negative rate. With strong discriminatory power, our random forest offers a cost-effective and non-invasive method to population-based screening for endometrial cancer prior to disease onset and is capable of targeting the sub-population with above-average risk. The ability to identify female patients with above-average risk can in turn inform the adoption of early cancer prevention strategies, including both immediate actions like screening and long-term preventative measures such as chemoprevention.

## Data Availability Statement

The data analyzed in this study was obtained from the NCI database https://cdas.cancer.gov/plco/ (PLCO-392). Requests to access the PLCO datasets should be directed to NCI Cancer Data Access System (CDAS) at cdas@imsweb.com.

## Author Contributions

GRH analyzed data, produced results, and wrote technical details and the manuscript. VY wrote parts of the manuscript and the code, generated results for a new RF algorithm, and responded to reviewers comments. GRH and VY made equal contributions to this work. GSH contributed to the study design, provided clinical consultation and interpretation of results, and wrote parts of the manuscript. YL did preliminary data exploration, provided technical consultation, and reviewed the manuscript. BN and WM provided technical consultation and reviewed the manuscript. JD generated research ideas, contributed to the study design, provided technical consultation, and reviewed the manuscript.

## Funding

This study was supported by a National Institutes of Health (NIH) National Institute of Biomedical Imaging and Bioengineering Award (R01EB022589) to JD. The content is solely the responsibility of the authors and does not necessarily represent the official views of the NIH.

## Conflict of Interest

The authors declare that the research was conducted in the absence of any commercial or financial relationships that could be construed as a potential conflict of interest.
